# Linking Single Domain Antibodies that Recognize Different Epitopes on the Same Target

**DOI:** 10.3390/bios2010043

**Published:** 2012-02-01

**Authors:** Richard H. Glaven, George P. Anderson, Dan Zabetakis, Jinny L. Liu, Nina C. Long, Ellen R. Goldman

**Affiliations:** 1 Nova Research Inc., 1900 Elkin Street, Suite 230, Alexandria, VA 22308, USA; E-Mails: rglaven@nmic.navy.mil (R.H.G.); nina.long.ctr@nrl.navy.mil (N.C.L.); 2 Center for Bio/Molecular Science and Engineering, Naval Research Laboratory, 4555 Overlook Ave. SW, Washington, DC 20375, USA; E-Mails: george.anderson@nrl.navy.mil (G.P.A.); daniel.zabetakis@nrl.navy.mil (D.Z.); jinny.liu@nrl.navy.mil (J.L.L.)

**Keywords:** avidity, single domain antibody, ricin detection

## Abstract

Single domain antibodies (sdAb) are the recombinantly expressed variable regions from the heavy-chain-only antibodies found in camelids and sharks. SdAb are able to bind antigens with high affinity, and most are capable of refolding after heat or chemical denaturation to bind antigen again. Starting with our previously isolated ricin binding sdAb determined to bind to four non-overlapping epitopes, we constructed a series of sdAb pairs, which were genetically linked through peptides of different length. We designed the series so that the sdAb are linked in both orientations with respect to the joining peptide. We confirmed that each of the sdAb in the constructs was able to bind to the ricin target, and have evidence that they are both binding ricin simultaneously. Through this work we determined that the order of genetically linked sdAb seems more important than the linker length. The genetically linked sdAb allowed for improved ricin detection with better limits of detection than the best anti-ricin monoclonal we evaluated, however they were not able to refold as well as unlinked component sdAb.

## 1. Introduction

Single domain antibodies (sdAb) are recombinant binding elements derived from the variable region of the heavy-chain-only antibodies found in camelids and sharks [1,2,3,4]. Like conventional antibodies, and their recombinantly derived binding elements (scFv), many sdAb bind their cognate antigen with high affinity and specificity [5,6]. The majority of sdAb, however, are stable; most are able to re-fold and bind antigen after heat or chemical denaturation, while conventional antibodies and scFv aggregate irreversibly under identical treatment [7,8,9]. While many sdAb have affinities measured to be sub-nanomolar, their expression as multimeric binding domains is a way of increasing the overall affinity of ligands that target epitopes close enough to allow two or more domains to bind simultaneously [10]. If more than one binding domain is bound to its respective epitope, the chances of both dissociating at the same time are much less than that of a single domain. Therefore, the off-rate is significantly lowered and the multimer’s effective affinity is elevated [11,12]. We hypothesized that genetically linking two sdAb that recognize different epitopes on the same target would provide reagents with improved utility for ricin detection, while maintaining the ability to refold demonstrated by the component sdAb.

Constructs in which scFv have been genetically linked to form bi-specific or bi-valent recognition elements have been described [13,14,15]. Bi-specific constructs link scFv specific for 2 targets, while bi-valent scFv target non-overlapping epitopes on the same antigen. Connecting two binders through a flexible peptide linker proved to be a strategy to design high affinity recognition reagents. The hen egg lysozyme (HEL) specific scFvs D1.3 (KD 1e−8 M) and HyHEL-10 (KD 1e−6 M) were joined by a flexible 15-mer linker to form a CRAb or chelating recombinant antibody [13]. The CRAb was shown to have a Kd of between 1e−9 and 1e−10 M by three different methods. The experimental data from the anti-HEL CRAb fit predictions generated by theory to explain the affinity enhancement shown by linked antibody fragments [12]. The degree of enhancement is dependent on factors including the affinities of the individual antibody fragments as well as the linker length. Wright and Deonarain [15] linked two anti- HEL scFv with a library of linkers varying in length and composition. Linker lengths in the library ranged from 11 to 54 amino acids; however the majority of selected clones had linkers of between 16 and 21 amino acids. If the linker was too short only one of the two scFvs was able to bind to the target at a time. However, excessively long linker lengths were found to be topologically and entropically unfavorable. 

Researchers have also linked sdAb to form bi-valent and bi-specific constructs [16,17,18,19,20]. Conrath *et al.* reported bi-specific constructs in which sdAb recognizing 2 different proteins were joined, and bi-valent sdAb in which two of the same sdAb were linked [16]. The bivalent sdAb constructs reported in that work showed a decrease in the off rate, leading to a 5-fold avidity enhancement. Additionally, they found position was of importance in their bi-specific constructs, with some sdAb showing lower affinity when on the C-terminal end. For example, when one of the anti-lysozyme sdAb clones was linked to a sdAb specific for Nmc-A (nonmetallo carbapenemase of class A), the measured on rate binding to lysozyme was 5 times slower for the bi-specific clone when the anti-lysozyme clone was on the C-terminal side of the linked construct as compared to the anti-lysozyme sdAb by itself or when on the N-terminal side of the linked construct. Other sdAb evaluated showed the same binding kinetics independent of their position in the constructs. Their constructs were shown to be stable on incubation at 37 °C for at least 44 h. 

More recently, Hultberg *et al.* linked sdAb specific for viral envelope proteins and reported dramatic improvements in neutralization potentials [20]. Llama-derived sdAb specific for Respiratory Syncytial Virus (RSV), Rabies virus and H5N1 Influenza were selected from immune libraries. The sdAb were joined through linkers consisting of (Gly_4_/Ser) repeats that were between 9 and 35 amino acids long, forming multivalent constructs in which two or three identical sdAb were linked. Additional studies evaluated bi-paratopic constructs, in which sdAb were joined that recognize different epitopes on the viral envelope proteins. In that work the authors found that in some cases linker length played a role in the viral neutralizing capability. For example, linker length did not seem important in bi-valent constructs targeting RSV; however, it did affect bi-paratopic constructs targeting the virus. While order of the sdAb within the construct was not extensively examined, it also seemed important; a bi-paratopic construct targeting RSV had a 15-fold difference in neutralizing ability depending on the order of the sdAb within the construct. 

Ricin-specific, llama-derived sdAb that recognize different epitopes on the toxin were the starting point for our linked sdAb constructs [21,22]. The parental sdAb recovered at least 50% of their secondary structure after heat denaturation. We prepared linked constructs in which two ricin-binding sdAb were joined via a flexible peptide, from 11 to 33 amino acid residues in length, and examined their binding to ricin and ability to re-fold after heat denaturation. The characteristics of the linked constructs were compared to sdAb and conventional antibodies. In this study sdAb order was more important than linker length in modulating the properties of the construct. We found that while the linked constructs provided improved limits of detection, they did not retain the ability to refold after heating as seen in the component sdAb.

## 2. Experimental Section

### 2.1. Reagents

Ricinus Communis Agglutinin II (ricin) and Ricinus Communis Agglutinin I (RCA120) were purchased from Vector (Burlingame, CA, USA). Abrin was from Toxin Technologies (Sarasota, FL, USA). The monoclonal antibodies (mAbs) 30-2C9 and 5F4 were provided by Tetracore (Rockville, MD, USA). PhycoLink^®^ Streptavidin-R-Phycoerythrin PJ31S (SA-PE) was obtained from Prozyme (San Leandro, CA, USA). Enzymes used for cloning were from New England Biolabs (Ipswich, MA, USA). Phosphate buffered saline (PBS), Tween 20, and bovine serum albumin (BSA) were obtained from Sigma-Aldrich (St. Louis, MO, USA). 

### 2.2. Preparing Linked Constructs

The ricin-binding llama sdAb utilized in this work were characterized and published previously [21,22]. Starting with the sdAb listed in Table 1, we designed a series of constructs in which sdAb were genetically linked through different length linkers (Table 2). Clones were named by listing the first sdAb, linker length, and second sdAb in the construct, so H1-11-B4 is the construct where the sdAb-H1 is joined to sdAb-B4 through the 11 amino acid linker.

**Table 1:** Affinities of parental sdAb towards ricin and RCA120.

Clone	Ricin KD (M) ^1^	RCA120 KD (M) ^1^	Putative Epitope
sdAb-B4	4 × 10^−^^9^	No binding	Ricin B chain
sdAb-H1	3 × 10^−^^10^	5 × 10^−^^9^	Ricin A/B interface
sdAb-C8	2 × 10^−^^11^	1.4 × 10^−^^9^	Ricin A chain
sdAb-D1	5 × 10^−^^10^	6 × 10^−^^9^	Ricin A chain

^1^ Determined by SPR [22].

**Table 2:** Linker lengths and sequences.

Linker length	Linker sequence
11	AAAGSGGASGS
16	AAAGSGSGGGSGASGS
21	AAAGSGSGGGSSGGGSGASGS
26	AAAGSGSGGGSSGGGSSGGGSGASGS
31	AAAGSGSGGGSSGGGSSGGGSSGGGSGASGS
33	AAAEPKIPQPQKPQPQPQPQPQQKPQQKPEPGS

Sequences in single amino acid code. 33 residue linker is derived from the natural upper hinge of llama IgG2 [16]. Linkers were designed so that AAA encodes a Not I restriction site and the C-terminal GS encodes a Bam HI site.

Using the previously determined sdAb amino acid sequences [21,22], inserts containing linked sdAb were synthesized by Genscript (Piscataway, NJ) with the *E. coli* codon usage optimized. The first sdAb in each construct was flanked by NcoI and NotI sites while the second sdAb was between BamHI and XhoI sites. We then used standard molecular cloning to mobilize the inserts containing linked sdAb into pet22b+ (Novagen) as NcoI-XhoI fragments and to construct several sets in which the same two sdAb were joined in each orientation through various linkers. Proteins were expressed and purified as described previously [23,24]. Expression levels of the linked constructs were comperable to the individual domains.

### 2.3. Preparation of Luminex Reagents and Assay Protocols

Luminex (Austin, TX) xMAP carboxylated microspheres were crosslinked to a variety of proteins using the two-step carbodiimide coupling protocol provided by the manufacturer. Direct binding and sandwich assays were performed essentially as described previously [21].

### 2.4. Circular Dichroism Measurements

CD measurements were performed using a Jasco J-815 CD spectropolarimeter equipped with a PTC-423S peltier temperature control system, as described previously [22]. Melting point data were acquired at a single wavelength between 202 and 210 nm, at a temperature rate of 5 °C/min over the range of 25 °C to at least 85 °C. We had previously shown that heating and cooling at rates of 1 °C/min or 5 °C/min yielded practically identical results [25]. Other researchers have evaluated the melting and refolding of sdAb using a heating and cooling rate as high as 10 °C/min [24].

### 2.5. SPR Kinetics Analysis

The SPR kinetic measurements were performed using the ProteON XPR36 (Bio-Rad) as described previously [22]. Briefly, a GLC chip was coated with the ricin (5 and 3 µg/mL) along with RCA 120 (5 and 3 µg/mL), ricin A chain (5 µg/mL) and commercial Abrin (5 µg/mL). The binding of the linked sdAb were tested by flowing 6 concentrations, from 100 nM to 0 nM, at 50 µL/min for 180 s over the antigen coated chip, and then monitoring dissociation for 900 s. The chip was regenerated using 50 mM glycince-HCl (pH 2.5) for 36 s, prior to any additional testing. The data were analyzed with the ProteON Manager TM 2.1 software; corrected by subtraction of the zero antibody concentration column as well as interspot corrected. The binding constants were determined using the software’s Langmuir model. The standard error of the affinity constants determination was typically less than 3% of the determined value.

## 3. Results and Discussion

### 3.1. Linked Constructs

We constructed and expressed genetically linked sdAb consisting of anti-ricin sdAb that recognize different epitopes on the toxin. The linkers used in the constructs ranged from 11 to 33 amino acids long (Table 2). SdAb were linked with different length Gly-Ser-type linkers commonly used in scFv constructs. In addition we included a linker with the sequence of the natural upper hinge of llama IgG2, used in the first report of linked sdAb; the upper hinge linker had originally been chosen for its flexibility and protease resistance [16]. Specifically, we examined constructs in which a ricin B chain binder (sdAb-B4) was joined to a binder that appears to bind an epitope that overlaps the ricin A and ricin B chain (sdAb-H1), constructs in which sdAb-H1 was linked to a ricin A chain binder (sdAb-C8), and constructs in which two A chain binders (sdAb-C8 and sdAb-D1) were linked. Clones were named by listing the first sdAb, linker length, and second sdAb in the construct. Although not all pairs were joined through the whole range of linkers, in each case we examined the effect of linker order on affinity and ability to refold after denaturation.

### 3.2. Determination of sdAb Function

**Figure 1:**
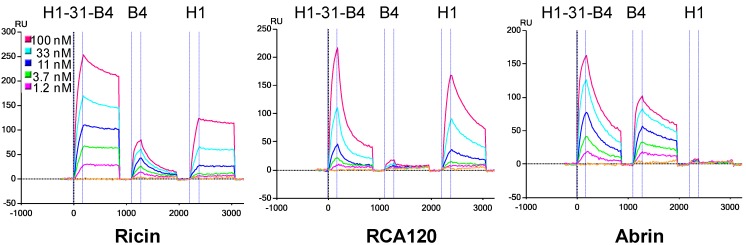
Binding of H1-31-B4, and parental sdAb-B4 and sdAb-H1 to ricin (**left**), RCA120 (**middle**) and abrin (**right**). The H1-31-B4 binds to all three targets, showing that each of the component sdAb is able to recognize target. The X axis is time in seconds, while the Y is the SPR signal reported in resonance units (RU). The different colored curves represent dilutions of the sdAb and linked construct as indicated in the figure. The vertical lines represent the beginning and ending of the injection of linked or parental sdAb.

After expressing the linked sdAb constructs, we used SPR to evaluate their binding specificities to ricin, RCA120, ricin A chain, and abrin. These targets allowed us to verify that each of the sdAb in the linked construct was functioning. For example, Figure 1 shows binding of H1-31-B4, along with its component sdAb, to ricin, RCA120, and abrin. RCA120 is a protein that shares high homology to ricin, but is much less toxic [26]. Abrin, like ricin, is a potent ribosome inhibiting toxin; the two toxins have high structural homology as well as some sequence homology [27,28,29]. The sdAb-H1 binds RCA120 but not abrin while sdAb-B4 binds abrin and not RCA120. The H1-31-B4 binds to RCA120 as well as to abrin, confirming that each of the sdAb in the linked construct was still able to bind target. Similarly, constructs consisting of the same sdAb in the opposite orientation as well as those containing different sdAb were evaluated to verify that each sdAb was able to bind target (data not shown).

Although both sdAb in the H1-B4 constructs are able to bind target, the data suggest that the sdAb within the linked constructs each bind with lower affinity than the unlinked component sdAb. As can be seen in Figure 1, the off rates of the linked construct on RCA120 and abrin are faster than the off rates of sdAb-H1 and sdAb-B4. Researchers characterizing bi-specific linked sdAb previously observed that sdAb within some of the linked construct had worse affinities than the unlinked sdAb [16].

### 3.3. Determination of sdAb Binding Target Simultaneously

**Figure 2:**
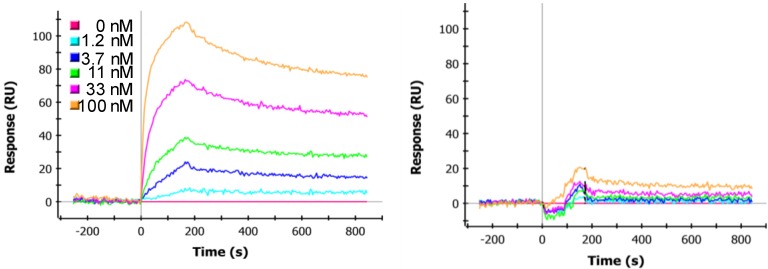
Sequential binding to ricin surface towards demonstrating simultaneous binding by sdAb in the H1-31-B4 construct. The left panel shows binding of a linked B4-31-B4 to a ricin surface that has been coated with sdAb-H1. The sdAb-H1 was applied over the ricin surface at a concentration of 100 nM for 3 min to approach saturation. Next the B4-31-B4 was applied in increasing concentrations as indicated on the figure. The right panel shows the B4-31-B4 applied to a ricin surface that had been coated with the H1-31-B4 construct to approach saturation using the same conditions as with the sdAb-H1. The same range of B4-31-B4 concentrations was applied.

It was more difficult to determine if both sdAb were binding target at the same time. Assays in which components were added to the ricin surface in series to see if binding was blocked seemed to indicate that, for at least the H1-B4 constructs, the sdAb are binding ricin simultaneously. As can be seen in Figure 1, the sdAb-B4 has a fast off rate when bound to a ricin surface. The fact that the off rate of sdAb-H1 and that of the linked construct are much slower indicates that the sdAb-H1 is binding to the ricin target. In order to do these experiments we prepared a construct in which B4 was linked to itself through the 31 amino acid linker, giving a slower off rate. Figure 2 shows that when the sdAb H1 is bound to the ricin surface that the B4-31-B4 construct can also bind, indicating that when the sdAb-H1 binds ricin, it does not block sdAb-B4’s epitope. The right panel of Figure 2 shows that when H1-31-B4 binds to the ricin surface, the binding of the B4-31-B4 construct is blocked, suggesting that the sdAb-B4 component of the linked construct is also actively binding target. These experiments, however, cannot distinguish if each sdAb in the linked constructs is binding to the same ricin, or neighboring ricins on the surface.

### 3.4. Effect of Linker Length and sdAb Order

We examined the binding data to determine the effect of linker length and sdAb order on the binding properties of the linked sdAb. 

**Table 3:** Affinity of linked constructs for ricin and RCA120.

	Affinity of linked constructs for ricin			Affinity of linked constructs for RCA 120		
Clone	ka (1/Ms) ^1^	kd (1/s) ^1^	KD (M) ^2^	ka (1/Ms) ^1^	kd (1/s) ^1^	KD (M) ^2^
H1-11-B4	2.2 × 10^5^	1.3 × 10^−^^4^	5.8 × 10^−^^10^	1.9 × 10^5^	1.6 × 10^−^^3^	8.7 × 10^−^^9^
H1-16-B4	2.4 × 10^5^	1.1 × 10^−^^4^	5.0 × 10^−^^10^	2.2 × 10^5^	1.8 × 10^−^^3^	7.9 × 10^−^^9^
H1-21-B4	2.4 × 10^5^	8.4 × 10^−^^5^	3.6 × 10^−^^10^	2.2 × 10^5^	1.8 × 10^−^^3^	8.4 × 10^−^^9^
H1-31-B4	2.1 × 10^5^	1.8 × 10^−^^4^	8.9 × 10^−^^10^	2.4 × 10^5^	3.7 × 10^−^^3^	1.6 × 10^−^^8^
H1-33-B4	2.2 × 10^5^	5.5 × 10^−^^5^	2.5 × 10^−^^10^	2.2 × 10^5^	2.0 × 10^−^^3^	9.1 × 10^−^^9^
C8-11-H1	5.3 × 10^5^	1.9 × 10^−^^5^	3.6 × 10^−^^11^	1.5 × 10^5^	3.3 × 10^−^^4^	2.1 × 10^−^^9^
C8-26-H1	4.9 × 10^5^	1.5 × 10^−^^5^	3.0 × 10^−^^11^	1.4 × 10^5^	1.9 × 10^−^^4^	1.3 × 10^−^^9^
B4-33-H1	2.0 × 10^5^	8.9 × 10^−^^4^	4.6 × 10^−^^9^	1.6 × 10^5^	3.0 × 10^−^^3^	1.9 × 10^−^^8^
H1-16-C8	1.0 × 10^5^	1.8 × 10^−^^5^	1.8 × 10^−^^10^	4.5 × 10^4^	1.2 × 10^−^^4^	2.6 × 10^−^^9^
C8-11-D1	7.9 × 10^5^	1.1 × 10^−^^4^	1.4 × 10^−^^10^	2.9 × 10^5^	1.9 × 10^−^^3^	6.6 × 10^−^^9^
D1-16-C8	8.4 × 10^5^	3.6 × 10^−^^5^	4.3 × 10^−^^11^	2.5 × 10^5^	2.5 × 10^−^^4^	9.9 × 10^−^^10^

^1^ Values determined by SPR. ^2^ KD calculated from ka and kd values.

As the data in Table 3 demonstrate, linker length appeared to have minimum effect on binding of H1-B4 linked constructs to ricin and RCA120. The C8-H1 linked constructs also showed essentially the same binding kinetics with two different linker lengths. The sdAb order, however, did appear important. For example, the off rate of H1-33-B4 is about 16 times slower than that of B4-33-H1. The order of the sdAb in the constructs linking sdAb-H1 and sdAb-B4 did not have as much of an effect on binding to RCA120. As sdAb-B4 does not bind RCA120, this suggests that the sdAb-H1 component binds RCA120 with nearly the same affinity in each position in the linked construct. The order of sdAb in linked constructs can impact their ability to bind target, and some sdAb have different affinities when they are in the first or second position [16].

The affinities of the linked constructs for ricin and RCA120 were compared to those of the parental sdAb (Table 1 and Table 3). In the H1- B4 series, the affinities for ricin and RCA120 were approximately the same as for the parental sdAb-H1. The C8- H1 constructs showed affinities essentially the same as the higher affinity sdAb, sdAb-C8. When sdAb-C8 and sdAb-D1were joined, the affinity for ricin and RCA120 was approximately the same as sdAb-C8, however the affinity for RCA120 dropped about 7-fold when the order of the sdAb in the construct was reversed.

### 3.5. Incorporation in Sandwich Assays for Ricin Detection

**Figure 3:**
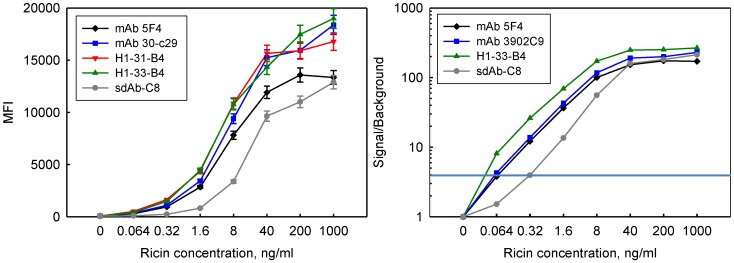
Fluid array assay for the detection of ricin using linked constructs, mAbs and an sdAb as capture reagents combined with a llama polyclonal anti-ricin reporter reagent. The linked constructs and mAbs gave similar absolute signals, whereas the sdAb gave lower signal. Error bars represent the variation between replicate experiments (**left**). The linked sdAb have lower background and perform better than the mAbs or sdAb when looking at signal *versus* background (**right**).

We incorporated the linked sdAb as capture elements in Luminex fluid array assays for the detection of ricin and compared limits of detection obtained using the new reagents to those using conventional mAbs and sdAb. Results are shown in Figure 3 for detection of ricin where linked sdAb along with two mAbs and an unlinked sdAb were immobilized on Luminex microspheres. Dilutions of ricin were applied to the microspheres and binding was detected through use of polyclonal llama anti-ricin antibody. The family of H1-B4 constructs all performed about equivalently in the detection of ricin and gave approximately the same magnitude of signal as the monoclonal antibodies. However, the linked constructs are very specific and have a much lower background signal than the mAbs in the absence of ricin. This can be seen when one plots signal over background. In repeated studies, these linked constructs provided for lower limits of detection than the mAbs. Setting threshold for detection at a signal to background ratio of 4, the linked constructs are able to detect at least 0.064 ng/mL ricin while the mAbs are reliably able to detect 0.32 ng/mL.

We had previously shown that sdAb-B4 immobilized on microspheres was not able to function as a captor in luminex sandwich assays [22], and that the sdAb-H1 does not perform as well as sdAb-C8 as a capture [21]. The sdAb-C8 was included as a sdAb benchmark and provided detection limits of 1.6 ng/mL, identical to what we previously reported [21]. 

### 3.6. Thermal Stability and Ability to Refold after Heat Denaturation

**Figure 4:**
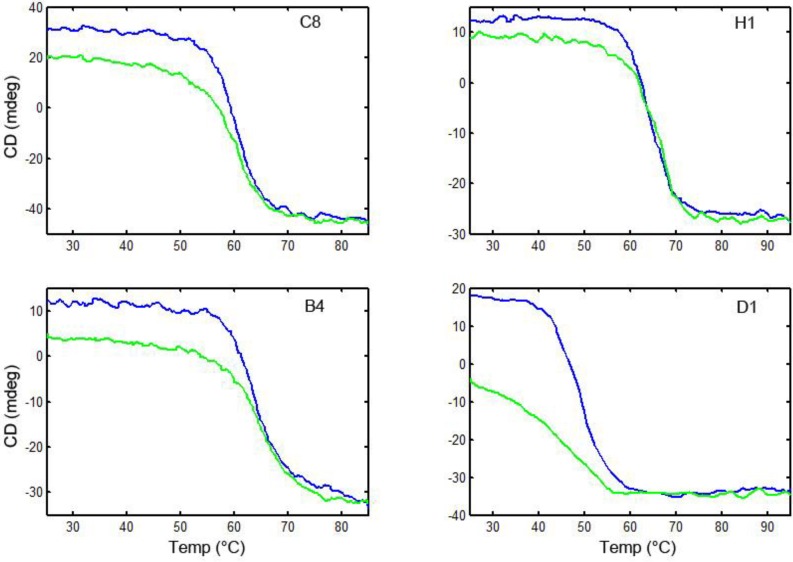
Circular dichroism of parental sdAb. Following the elipticity of the sdAb on heating and then cooling, the melting and subsequent refolding of these sdAb can be monitored. Blue curve is heating, and green is cooling.

**Figure 5:**
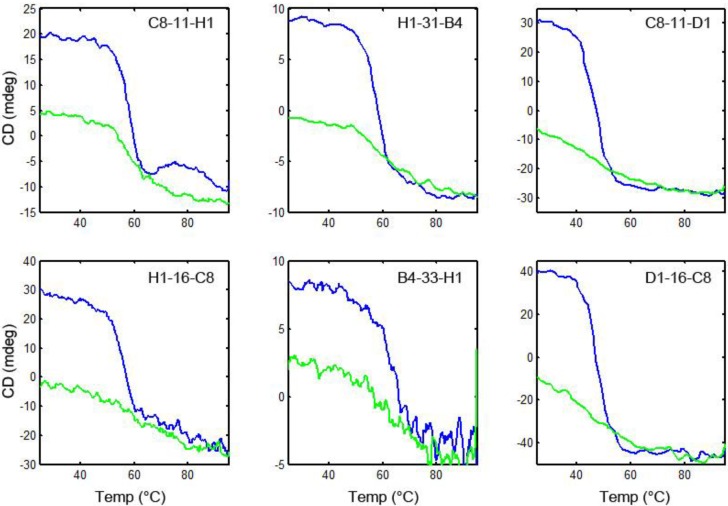
Circular dichroism of linked constructs. Representative constructs in which sdAb are joined in each orientation are shown. Blue curve is heating, and green is cooling.

To evaluate the melting temperature of the linked sdAb and their ability to re-fold after heat denaturation, we used circular dichroism (CD). First, we confirmed that the component, unlinked sdAb all unfolded on heating and then re-folded when cooled (Figure 4). Then each of the linked sdAb was evaluated to determine its melting temperature and ability to re-gain its secondary structure after heating. Representative spectra are shown in Figure 5; none of the linked sdAb constructs re folded as well as the component sdAb. We had previously demonstrated the ability of the component sdAb to function in assays for the detection of ricin after heat denaturation, and found that there was a good correlation between the recovery of secondary structure as judged by the CD and the ability of the sdAb to function after heating [21,22]. From the CD data, all of the linked constructs appeared to re-fold poorly after denaturation; we did not test their binding ability after heating as we expected them to have lost a large percentage of their binding ability.

## 4. Conclusions

By examining the binding of the linked constructs to several immobilized proteins, it was fairly straight forward to determine if each of the sdAb in the linked construct was able to bind target. Determining if the sdAb in the linked construct were binding simultaneously was more challenging. As the starting sdAb all have sub nM affinity for target; it was difficult to use the SPR to differentiate off rates for antibody fragments that come off the surface slowly. Factors such as instrument baseline drift can limit one’s ability to discriminate difference in these high affinity ranges. Linking sdAb with poorer affinities could provide a means to evaluate the simultaneous binding of the components, but may not result in as useful recognition reagents.

We did not see an effect of linker length on properties of the linked anti-ricin sdAb. This suggests that the epitopes may be located close enough that the examined linker lengths do not impact the ability of the constructs to bind. It is possible that linker length might have been important, for example, when linking a ricin A chain binding sdAb such as sdAb-C8 to the ricin B chain binding sdAb-B4. The order of the sdAb within the linked construct, however, did appear to impact the properties of the constructs. It is not surprising that sdAb order within the linked structures made a difference in their binding kinetics, and is consistent with other reports [16,20]. In the linked constructs, the C-terminus of the first sdAb is linked to the N-terminus of the second sdAb; thus, the sterospecific binding sites of the linked sdAb are unlikely to be equivalent for sdAb (A-B) and (B-A). Another consideration is that potentially the linker can play a role in obstructing the binding of the complementarity determining regions (CDRs) of the second sdAb to its epitope.

Linking sdAb provided reagents that allowed for lower detection limits, however, the resulting constructs did not have the stability of the parental sdAb. Perhaps the close proximity of the sdAb in the linked constructs causes them to miss-fold after denaturation. Although unlike the component VH and VL that make up a conventional scFv, the sdAb are highly soluble independently. Future efforts will need to focus on ways to modify linked constructs to enhance refolding. Perhaps adding disulfides to make the linker more rigid and thus keep the component sdAb apart from each other will provide reagents that are able to refold after denaturation.

Another way to link antibody fragments, such as sdAb, is through multimerization domains. Pentameric sdAb constructs in which sdAb were expressed as fusions with an oligomerization domain have been described [30]. In that work, sdAb were fused with the B sub unit of *E. coli* verotoxin resulting in self-assembled pentameric binding entities in which each of the sdAb in the pentamer is identical. An increase in functional affinity of several orders of magnitude for immobilized antigen was realized using those constructs. The pentamers were demonstrated to be thermal stable and resistant to proteases. Homodimers, in which sdAb are linked to an *E. coli* alkaline phosphatase also provide high melting point constructs which serve to increase the functional affinity of sdAb-based reagents [31,32]. However, those constructs also consist of repeats of the same sdAb. An alternate structure in which sdAb are expressed at both the N and C terminal of the pentamerization domain resulted in bispecific decavalent molecules [33]. When constructing the bispecific decavalent constructs 4 different linkers were examined between the verotoxin protein and the sdAb fused at each end, and the linker identity was found to be important in the production of non-aggregated protein. 

Genetically linking sdAb provides an avenue for recognition reagents that yield improved limits of detection. Although these reagents do not refold as well as the component sdAb, they do regain a portion of their secondary structure, and provide a starting point for the development of more stable and re-foldable linked constructs. The use of multimerization domains provides another route for linked sdAb that may be thermal stable.

## References

[1] Ghahroudi M.A., Desmyter A., Wyns L., Hamers R., Muyldermans S. (1997). Selection and identification of single domain antibody fragments from camel heavy-chain antibodies. FEBS Lett..

[2] Greenberg A.S., Avila D., Hughes M., Hughes A., McKinney E.C., Flajnik M.F. (1995). A new antigen receptor gene family that undergoes rearrangement and extensive somatic diversification in sharks. Nature.

[3] Hamerscasterman C., Atarhouch T., Muyldermans S., Robinson G., Hamers C., Songa E.B., Bendahman N., Hamers R. (1993). Naturally-occurring antibodies devoid of light-chains. Nature.

[4] Nuttall S.D., Krishnan U.V., Hattarki M., de Gori R., Irving R.A., Hudson P.J. (2001). Isolation of the new antigen receptor from wobbegong sharks, and use as a scaffold for the display of protein loop libraries. Mol. Immunol..

[5] Wesolowski J., Alzogaray V., Reyelt J., Unger M., Juarez K., Urrutia M., Cauerhff A., Danquah W., Rissiek B., Scheuplein F., Schwarz N., Adriouch S., Boyer O., Seman M., Licea A., Serreze D.V., Goldbaum F.A., Haag F., Koch-Nolte F. (2009). Single domain antibodies: Promising experimental and therapeutic tools in infection and immunity. Med. Microbiol. Immunol..

[6] De Marco A. (2011). Biotechnological applications of recombinant single-domain antibody fragments. Microb. Cell Factories.

[7] Van der Linden R.H.J., Frenken L.G.J., de Geus B., Harmsen M.M., Ruuls R.C., Stok W., de Ron L., Wilson S., Davis P., Verrips C.T. (1999). Comparison of physical chemical properties of llama V-HH antibody fragments and mouse monoclonal antibodies. Biochim. Biophys. Acta.

[8] Perez J.M.J., Renisio J.G., Prompers J.J., van Platerink C.J., Cambillau C., Darbon H., Frenken L.G.J. (2001). Thermal unfolding of a llama antibody fragment: A two-state reversible process. Biochemistry.

[9] Ewert S., Cambillau C., Conrath K., Pluckthun A. (2002). Biophysical properties of camelid V-HH domains compared to those of human V_H_3 domains. Biochemistry.

[10] Pluckthun A., Pack P. (1997). New protein engineering approaches to multivalent and bispecific antibody fragments. Immunotechnology.

[11] Crothers D.M., Metzger H. (1972). Influence of polyvalency on binding properties of antibodies. Immunochemistry.

[12] Zhou H.X. (2003). Quantitative account of the enhanced affinity of two linked scFvs specific for different epitopes on the same antigen. J. Mol. Biol..

[13] Neri D., Momo M., Prospero T., Winter G. (1995). High affinity antigen-ginding by chelating-recombinant-antibodies (CRABS). J. Mol. Biol..

[14] Korn T., Nettelbeck D.M., Volkel T., Muller R., Kontermann R.E. (2004). Recombinant bispecific antibodies for the targeting of adenoviruses to CEA-expressing tumour cells: A comparative analysis of bacterially expressed single-chain diabody and tandem scFv. J. Gene Med..

[15] Wright M.J., Deonarain M.P. (2007). Phage display of chelating recombinant antibody libraries. Mol. Immunol..

[16] Conrath K.E., Lauwereys M., Wyns L., Muyldermans S. (2001). Camel single-domain antibodies as modular building units in bispecific and bivalent antibody constructs. J. Biol. Chem..

[17] Coppieters K., Dreier T., Silence K., de Haard H., Lauwereys M., Casteels P., Beirnaert E., Jonckheere H., de Wiele C.V., Staelens L., Hostens J., Revets H., Remaut E., Elewaut D., Rottiers P. (2006). Formatted anti-tumor necrosis factor alpha VHH proteins derived from camelids show superior potency and targeting to inflamed joints in a murine model of collagen-induced arthritis. Arthritis Rheum..

[18] Hmila I., Abdallah B.A.B., Saerens D., Benlasfar Z., Conrath K., El Ayeb M., Muyldermans S., Bouhaouala-Zahar B. (2008). VHH, bivalent domains and chimeric Heavy chain-only antibodies with high neutralizing efficacy for scorpion toxin AahI’. Mol. Immunol..

[19] Simmons D.P., Abregu F.A., Krishnan U.V., Proll D.F., Streltsov V.A., Doughty L., Hattarki M.K., Nuttall S.D. (2006). Dimerisation strategies for shark IgNAR single domain antibody fragments. J. Immunol. Methods.

[20] Hultberg A., Temperton N.J., Rosseels V., Koenders M., Gonzalez-Pajuelo M., Schepens B., Ibanez L. I., Vanlandschoot P., Schillemans J., Saunders M., Weiss R.A., Saelens X., Melero J.A., Verrips C.T., Van Gucht S., de Haard H.J. (2011). Llama-derived single domain antibodies to build multivalent, superpotent and broadened neutralizing anti-viral molecules. PLoS One.

[21] Anderson G.P., Liu J.L., Hale M.L., Bernstein R.D., Moore M., Swain M.D., Goldman E.R. (2008). Development of antiricin single domain antibodies toward detection and therapeutic reagents. Anal. Chem..

[22] Anderson G.P., Bernstein R.D., Swain M.D., Zabetakis D., Goldman E.R. (2010). Binding kinetics of antiricin single domain antibodies and improved detection using a B chain specific binder. Anal. Chem..

[23] Goldman E.R., Anderson G.P., Liu J.L., Delehanty J.B., Sherwood L.J., Osborn L.E., Cummins L.B., Hayhurst A. (2006). Facile generation of heat-stable antiviral and antitoxin single domain antibodies from a semisynthetic llama library. Anal. Chem..

[24] Conway J.O., Sherwood L.J., Collazo M.T., Garza J.A., Hayhurst A. (2010). Llama single domain antibodies specific for the 7 botulinum neurotoxin serotypes as heptaplex immunoreagents. PLoS One.

[25] Anderson G.P., Zabetakis D., Bernstein R.D., Cai S.W., Singh B.R., Goldman E.R. (2011). Evaluation of anti-hemagglutinin Hn-33 single domain antibodies: Kinetics, binding epitopes, and thermal stability. Botulinum J..

[26] Roberts L.M., Lamb F.I., Pappin D.J.C., Lord J.M. (1985). The primary sequence of ricin-communis agglutinin—Comparison with ricin. J. Biol. Chem..

[27] Kimura M., Sumizawa T., Funatsu G. (1993). The complete amino-acid sequences of the B-chains of abrin-A and abrin-G, toxic proteins from the seeds of *Abrus-precatorius*. Biosci. Biotechnol. Biochem..

[28] Wood K.A., Lord J.M., Wawrzynczak E.J., Piatak M. (1991). Preproabrin—Genomic cloning, characterization and the expressin of the A-chain in *Escherichia-coli*. Eur. J. Biochem..

[29] Robertus J.D., Monzingo A.F. (2004). The structure of ribosome inactivating proteins. Mini Rev. Med. Chem..

[30] Zhang J.B., Li Q.G., Nguyen T.D., Tremblay T.L., Stone E., To R., Kelly J., MacKenzie C.R. (2004). A pentavalent single-domain antibody approach to tumor antigen discovery and the development of novel proteomics reagents. J. Mol. Biol..

[31] Sherwood L.J., Osborn L.E., Carrion R., Patterson J.L., Hayhurst A. (2007). Rapid assembly of sensitive antigen-capture assays for Marburg virus, using *in vitro* selection of llama single-domain antibodies, at biosafety level 4. J. Infect. Dis..

[32] Swain M.D., Anderson G.P., Serrano-González J., Liu J.L., Zabatakis D., Goldman E.R. (2011). Immunodiagnostic reagents using llama single domn antibody-alkaline phosphatase fusion proteins. Anal. Biochem..

[33] Stone E., Hirama T., Tanha J., Tong-Sevinc H., Li S.H., MacKenzie C.R., Zhang J.B. (2007). The assembly of single domain antibodies into bispecific decavalent molecules. J. Immunol. Methods.

